# Exploring the Potential of Laser Ablation Carbon Isotope Analysis for Examining Ecology during the Ontogeny of Middle Pleistocene Hominins from Sima de los Huesos (Northern Spain)

**DOI:** 10.1371/journal.pone.0142895

**Published:** 2015-12-16

**Authors:** Nuria Garcia, Robert S. Feranec, Benjamin H. Passey, Thure E. Cerling, Juan Luis Arsuaga

**Affiliations:** 1 Departamento de Paleontologia, Facultad de Ciencias Geológicas, Universidad Complutense de Madrid, Ciudad Universitaria 28040 Madrid, Spain; 2 Centro Mixto (UCM-ISCIII) de Evolución y Comportamiento Humanos, C/Monforte de Lemos 2–4, Pab. 14, 28029 Madrid, Spain; 3 Research and Collections Division, New York State Museum, 3140 Cultural Education Center, Albany, NY 12230–0001, United States of America; 4 Department of Earth and Planetary Sciences, Johns Hopkins University, 120 Olin Hall, 3400 N. Charles Street, Baltimore, MD 21218, United States of America; 5 Department of Geology & Geophysics, University of Utah, Frederick Albert Sutton Building, 115 S 1460 East Room 383, Salt Lake City, UT 84112, United States of America; Museo Nazionale Preistorico Etnografico 'L. Pigorini', ITALY

## Abstract

Laser ablation of tooth enamel was used to analyze stable carbon isotope compositions of teeth of hominins, red deer, and bears from middle Pleistocene sites in the Sierra de Atapuerca in northern Spain, to investigate the possibility that this technique could be used as an additional tool to identify periods of physiological change that are not detectable as changes in tooth morphology. Most of the specimens were found to have minimal intra-tooth variation in carbon isotopes (< 2.3‰), suggesting isotopically uniform diets through time and revealing no obvious periods of physiological change. However, one of the two sampled hominin teeth displayed a temporal carbon isotope shift (3.2‰) that was significantly greater than observed for co-occurring specimens. The δ^13^C value of this individual averaged about -16‰ early in life, and -13‰ later in life. This isotopic change occurred on the canine crown about 4.2 mm from the root, which corresponds to an approximate age of two to four years old in modern humans. Our dataset is perforce small owing to the precious nature of hominid teeth, but it demonstrates the potential utility of the intra-tooth isotope profile method for extracting ontogenetic histories of human ancestors.

## Introduction

The hominins recovered from the Sima de los Huesos (SH) in the Sierra de Atapuerca (Spain) represent a majority (>80%) of the global fossil record for the genus *Homo* in the middle Pleistocene. At least 28 individuals of this species have been recovered at this locality, which is dated to about 430,000 years ago. The SH specimens show a mosaic pattern of cranial and mandibular features which fit the prediction of the accretion model for the first stage of Neandertal evolution [1–3]. The abundance of hominin specimens here (Number of Identified Specimens = >6500) also provides a unique opportunity to examine specific aspects of hominin development and ecology during the middle Pleistocene. For example, topics such as the timing of behavioral or developmental changes within a hominin population may be addressed, while there may be little power to elucidate such subjects at sites with more limited hominin remains.

Ascertaining when significant changes are concentrated during development can help determine periods critical for individual survival. For example, the timing of weaning may affect spacing between births and thus overall fertility [4–6]. However, precise identification of significant changes in physiology can be difficult as the changes must affect tissue growth and result in observable morphological variations, which does not always occur [7]. The timing of a physiological change may occur before or after the development of a particular morphological feature, or may be so strong that it results in the death of the individual before it can be recorded in the developing tissues [7]. To be identified morphologically, the physiological change must necessarily occur during the development of a particular feature and be of an appropriate strength to disrupt normal growth. While certain changes may not be of an appropriate strength to disrupt growth and become evident in the morphology, this does not necessitate that it is not recorded within the individual at all. For example, the change can be recorded in individuals at the chemical (i.e., isotopic) level, particularly if it affects or changes ingestion of food and water.

Dietary shifts may result in a significant change in the isotopic composition of animal tissues. Because stable isotopes are integrated into animal tissues based on what is ingested [8–10], many studies have used isotopic analysis to identify variation in diet within and among individuals [11–19]. Thus, stable isotope analysis provides a way to examine changes in food and water intake and may be useful as an additional tool to identify periods of significant physiological change for individuals within a population.

Identifying the physiological status of individuals in the SH hominin population has been a topic in prior studies [20–21]. For the most part, the data from these studies suggest that this population experienced limited restriction in the development of tooth enamel. Specifically, Cunha et al. (2004) [21] assessed the physiological status of this population by observing the incidence of enamel hypoplasias on teeth [21]. Enamel hypoplasias are modifications of the tooth crown surface due to enamel formation disruption that are the result of physiological changes that occur during tooth development. These researchers identified hypoplasias in less than 5% of the analyzed teeth with incisors and canines being the most affected. Further, the majority of hypoplasias were calculated to have occurred during the third year of life, possibly relating to the dietary shift associated with weaning [21].

The low percentage of hypoplasias in the SH hominin sample may be the result of abundant resources permitting relatively unhindered skeletal growth within the population, or, as mentioned above, due to differences in the timing or intensity of physiological changes, for example. Within this study, we explore the possibility that stable isotope analysis can identify significant changes in diet, and, subsequently, whether these changes can be related to particular ontogenetic events (e.g., weaning) as was suggested for the majority of hypoplasias in the SH hominins [21].

## Background and Methods

### Locality Information

**Fig 1:**
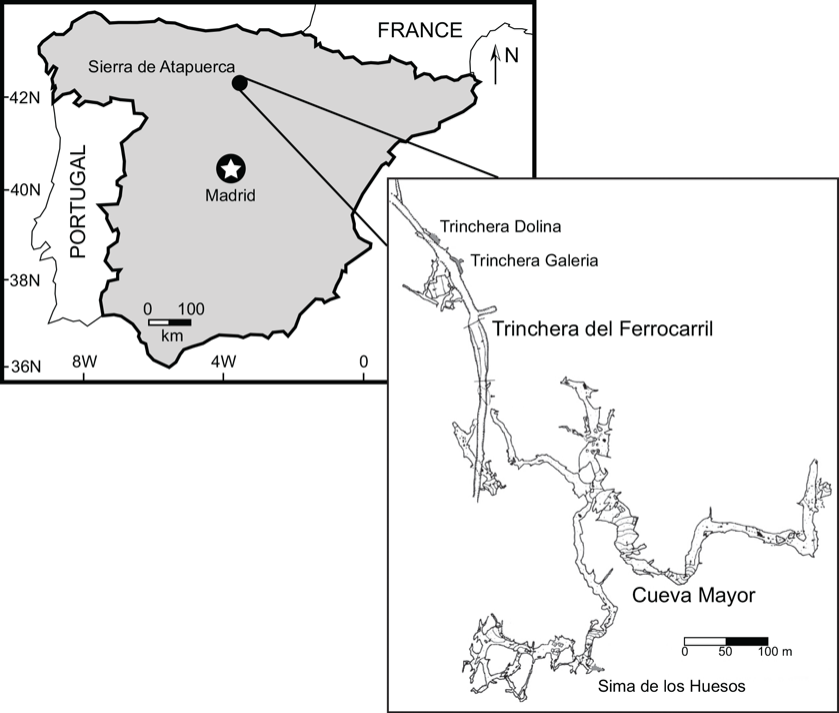
Location of the Sima de los Huesos in northern Spain. Inset map shows the three localities from which specimens derive (i.e., Trinchera Dolina, Trinchera Galería, Sima de los Huesos) highlighted in gray.

The SH site lies inside Cueva Mayor within the Sierra de Atapuerca in northern Spain (Fig 1). This site contains an extraordinary accumulation of approximately 28 hominin individuals in an ancient mud-breccia, along with a large assemblage of carnivores, particularly bears [2, 22–23], and sparse insectivore and rodent remains [24–25]. Over the last several years, attempts to establish an age for the hominin fossils within the Sima de los Huesos have yielded a number of dates. A minimum date of around 320 ka was proposed using electron spin resonance–uranium series (ESR/U-series; [26]). In a later study, U-series dating of a speleothem, interpreted as having formed after the hominin bone accumulation, yielded an age in excess of 530 ka [27]. Biostratigraphically, macro- and micro-faunal content of the deposits at SH correlate with layers from other Atapuerca sites that have been dated to the mid-Middle Pleistocene (c. 600–340 ka; [28]). More recently, genetic estimates of the age of a SH bear specimen based on the length of its mtDNA branch vary between 150–640 ka with point estimates close to 400 ka [29]. Further, an almost complete mitochondrial genome sequence of a hominin from SH has additionally been determined [30]. Based on the length of its mtDNA branch, the age of the SH hominin was also estimated to be between 150–640 ka with point estimates close to 400 ka, in exact agreement with the point estimate for the *U*. *deningeri* mtDNA. Using the above and a suite of independent dating methods, an age of about 430 ka for the Sima de los Huesos hominin and bear accumulation has been established [3]. This age and the taxa contained in SH are roughly contemporaneous with particular levels at other nearby localities within the Sierra, such as Trinchera Dolina 10 (TD 10) and Trinchera Galería Units II and III (TG-II, TG-III), and all within Atapuerca-Faunal Unit 6 (Ata-FU-6), [3, 31–33].

### Sampling: Bulk Sampling and Laser Ablation

**Fig 2:**
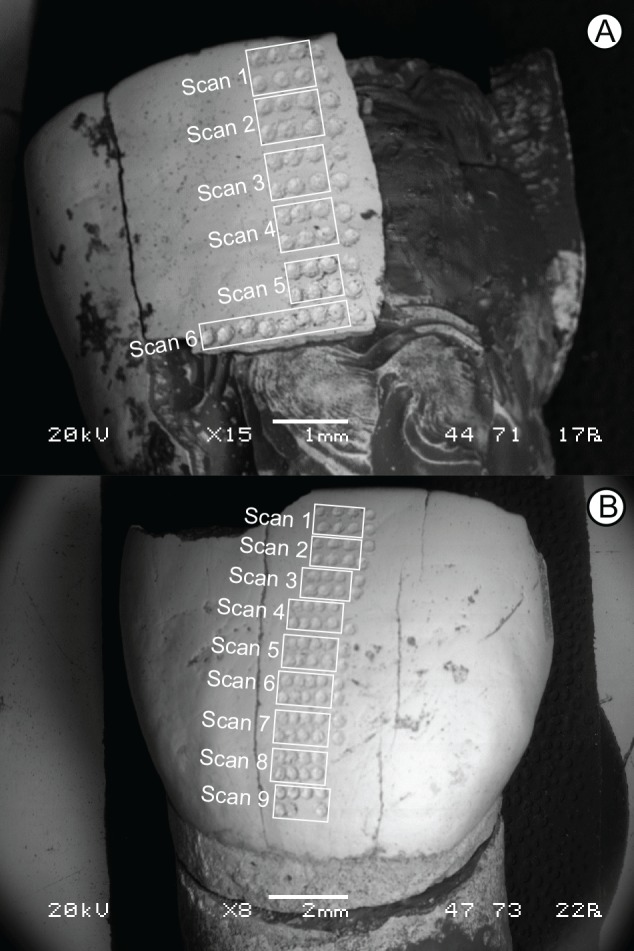
Photograph of laser ablation scans on the hominin teeth. A, canine AT-825; scans begin at the occlusal surface and are composed of six to eight total laser shots (four shots in two rows), except for the last scan, which is composed of nine shots in a single row. B, incisor AT-146; scans begin at the occlusal surface and are composed of six to eight shots (three to four shots in two rows). Shots from the single vertical scan (11 shots for AT-825, 13 for AT-146) are visible on the both hominin teeth to the right of horizontal scans on each photo.

There are a number of techniques used to obtain stable isotope data to address questions related to animal diet and dietary change. These include analyzing a single bulk sample from each individual tooth, or analyzing multiple samples from the same tooth to form a time-series of isotopic variation. Bulk sampling provides an average value for resource use during the time spanned by the development of the analyzed tooth, while intra-tooth sampling provides finer temporal detail than that obtained from bulk sampling [14,34–38]. Typically, intra-tooth sampling involves drilling numerous samples up the growth axis of the tooth crown, using a dental-style drill and drill bit. Sampling using this method leaves behind drill pits that are typically about 1 mm wide, 1–3 mm long, and 1mm deep, a tolerable level of damage for large or common fossil teeth, but generally prohibitive for hominin teeth. Here we utilize conventional bulk sampling as well as laser ablation GC/IRMS (Gas Chromatography/ Isotope Ratio Mass Spectrometry) to obtain isotope values. The laser ablation GC/IRMS technique can resolve carbon isotope values at a much finer scale than the typical dental-drilling method. The samples from the laser (herein called, “scans”) consist of a number of individual laser ablation events (“shots”) which create CO_2_ by thermal decarbonation of carbonate in the apatite mineral. In this study, each shot has a diameter of ~0.3 mm, and sequential scans can be much less than 1 mm apart (Fig 2). The number of shots used per scan varied among the analyzed samples from 6 to 25 shots, as required to generate enough pooled CO_2_ for isotopic analysis. While not absent, the level of surface damage from the laser is significantly less than that produced using a dental drill, and the ablation pits from each “shot” are less than ~100 microns deep, meaning that a volumetrically insignificant fraction of tooth enamel is removed for analysis. For this study, two hominin teeth from the SH site were analyzed, including one permanent upper right first incisor and one permanent upper left canine (Fig 2), both unassociated with a particular skull or individual. These specimens are in the collections of the Centro Mixto (UCM-ISCIII) de Evolución y Comportamiento Humanos under the scientific care and management of the corresponding author (J L Arsuaga), as such, special permits were not needed to sample these middle Pleistocene aged hominins. Requests regarding the availability of these samples for analysis should be sent to the corresponding author (J L Arsuaga), who is the current curator and directs the research on the SH hominin fossils. For comparison, we also analyzed three bear (*Ursus deningeri*) and two red deer (*Cervus elaphus*) specimens. As mentioned above, the SH site contains no herbivore specimens. The analyzed red deer specimens come from contemporaneous levels within the Trinchera Galería and Trinchera Dolina localities [3,31–33], located only a few hundred meters away from SH (Fig 1). Red deer are generalist herbivores [31, 39–40], and were chosen for analysis as they should provide an understanding of what type of isotopic variation is available for taxa. Similarly, bears were chosen as a faunal comparison to the hominin sample due to their abundance in the Sima. Further, because hibernation may have an effect on isotope values in bears [41–43], these samples may show how isotopic values change in relation to metabolic changes, such as those observed as the result of hibernation.

The laser analyses were conducted within the Department of Geology and Geophysics at the University of Utah. Before analysis, teeth were cleaned both mechanically and with acetone, and then dried. With samples inside, the laser chamber was purged with helium for several minutes or hours as required for the rate of CO_2_ outgassing to fall below appropriate levels. Small amounts (10–30 nmol) of CO_2_ were generated using a CO_2_ laser (10.6 micrometers) operating at 5–15 W and 8.5 ms pulse duration in a He atmosphere. The CO_2_ was cryogenically purified and ‘focused’ prior to introduction to a continuous-flow GC-IRMS (MAT 252). Systematic isotope fractionation and fractionation associated with laser ablation production of CO_2_ were monitored by analyses of injected aliquots of CO_2_, each calibrated against NBS-19 gas (δ^13^C = 1.95‰). Precision for the CO_2_ injections was 0.3‰ (Appendix B in S1 File). Laser scans were made perpendicular to the growth axis of the tooth for all analyzed teeth (Fig 2). Additionally, a vertical scan was taken on the sampled hominin and bear teeth. During our analyses, three scans were excluded. When conducting scan number 4 for the hominin sample, AT-146, we noticed the production of smoke (scan 4) and significant charred carbon residue (scan 9). Additionally, significant charred carbon residue remained after scan number 4 for the bear sample, SH-97 U14-137. Previous studies [38, 44] have shown that significant charring and/or smoke during the laser sampling indicates pyrolysis or partial combustion of organic compounds that produces isotopically anomalous CO_2_ that interferes with isotopic analysis. Because of this, we exclude these three analyses from the final dataset.

For comparison to the laser scans as well as comparison to data collected in a previous study analyzing isotope values from non-hominin fauna from the same faunal unit (Ata-FU-6; [31]), two bulk samples were taken, one from each of the hominin teeth, to allow comparison with the laser data. For AT-825, the bulk sample was taken along a fracture surface having thick enamel (i.e., away from the neck) in an area roughly corresponding to laser scans 3–6 (Fig 2A). Similarly, the bulk sample for AT-146 was taken along a fracture surface in the medial portion of the crown, roughly corresponding to laser scans 3–7 (Fig 2B). For these, sampling and preparation followed standard techniques [45–46]. Briefly, the fracture surfaces of each tooth were mechanically cleaned using a dental drill. About 5 mg of enamel powder was drilled from the clean enamel surface. This enamel was treated with 30% hydrogen peroxide to remove organics. The hydrogen peroxide was then decanted and the powder was washed with Milli–Q water, and then treated in 0.1N acetic acid to remove any diagenetic carbonates. The following day the acetic acid was decanted and the enamel powder was washed with Milli-Q water and air dried. These purified enamel samples were analyzed using an ISOCARB automated carbonate preparation system attached to a Micromass Optima gas source mass spectrometer within the Geology Department at the University of California, Davis. Samples were dissolved in 100% phosphoric acid at 90°C to create CO_2_. These samples were corrected to NBS-19 and UCD-SM92 an in-house marble standard. Precision for the two bulk samples was < 0.1‰ for carbon. Additionally, to better understand the ability to integrate bulk sampling carbon isotope values (conventional phosphoric acid method) to those obtained by laser ablation, we compare the laser results, including the horizontal serial scans and the vertical scan, to bulk sampling data from the same specimens presented here (i.e., for hominins) or from Garcia-Garcia et al. (i.e., for non-hominin fauna;[31]).

### Carbon Isotopes in Mammals

The carbon isotope results in this study are expressed in the standard δ-notation: δ^13^C = [((^13^C/^12^C)_sample_/(^13^C/^12^C)_standard_) - 1] × 1000. The δ^13^C values are reported relative to the Vienna-Pee Dee Belemnite (V-PDB) standard.

Studies analyzing carbon isotope data to infer diets or dietary change are useful because the different photosynthetic pathways that are used by plants (i.e., C_3_, C_4_, and Crassulacean Acid Metabolism (CAM)) impart different carbon isotope ratios to plant tissues, and animals consuming those plant tissues will reflect the ratio ingested [8–10]. Because of a concern regarding diagenetic alteration of isotope values in certain mammal tissues, this study focuses on analyzing the stable carbon isotope values from tooth enamel. While the effects of diagenesis may be a concern when examining some fossil tissues for stable isotope values [47], such as bone apatite (although see [48]), many studies over more the last 25 years have shown that tooth enamel is generally a faithful recorder of original δ^13^C values [46, 49–52]. Animal tissues reflect the carbon isotope compositions of consumed plants; tooth enamel is enriched in ^13^C relative to food by a consistent factor of 12 to 14‰, and as protein increases in the diet the enrichment may become smaller [53–54]. For example, C_3_ plants, which include most trees, shrubs, and cool-growing-season grasses, are relatively enriched in the light carbon isotope (^12^C), have a mean δ^13^C value of –27.0‰ and typically range from –22‰ to -35‰ [55–58]. Based on an enrichment factor of +13‰ and an average C_3_ dietary isotopic composition of -27‰, hominins with C_3_ diets should have tooth enamel δ^13^C values that average -14‰.

Based on the isotopic values of the modern Spanish flora as well as the data from a previous study that included fauna from the SH site [31], it is known that the vegetation around this locality was dominated by C_3_ plants [31, 59–61]. While many studies have used the isotopic differences between C_3_ and C_4_ plants to understand ecology, different processes (e.g., temperature variation, water stress), can produce variation in the δ^13^C value in C_3_ plants [56–57, 62–63]. Prior studies show that C_3_ plants generally have more negative carbon isotope values in closed, forested habitats, while plants in open and drier habitats generally have more positive isotope values [56, 58, 62–65]. Studies have used these differences in plant isotope values in C_3_-dominated environments to recognize dietary differences among species [31, 63, 65–70].

Relevant to investigating diet and dietary change in this study are temporal and spatial variations in the carbon isotope values of plants [55, 62, 71–73]. Across communities within ecosystems, carbon isotope values may vary. However, at a particular locality, the carbon isotope values of plants using a particular photosynthetic pathway do not appear to have a large total variation [55, 62, 71–73]. Within a population the total variation in carbon isotope values is generally less than 3‰ [55, 62, 71–73]. Similarly, intra-population δ^13^C variation due to seasonal changes is usually less than 1‰ [55, 62, 71–73]. Because of the limited intra-population variation in δ^13^C value at a particular locality even over different seasons, variation in carbon isotope values greater than 3.0‰ may indicate that an individual was eating different resources. An abrupt 3‰ change in δ^13^C value of tooth enamel should represent a conservative value for identifying significant changes in diet as the process of enamel mineralization and maturation tends to dampen the isotope signal (S1 File; [38, 54, 74]). Thus, a 3‰ difference observed in enamel δ^13^C values actually represents a much greater change in the primary isotope signal.

## Results and Discussion

**Fig 3:**
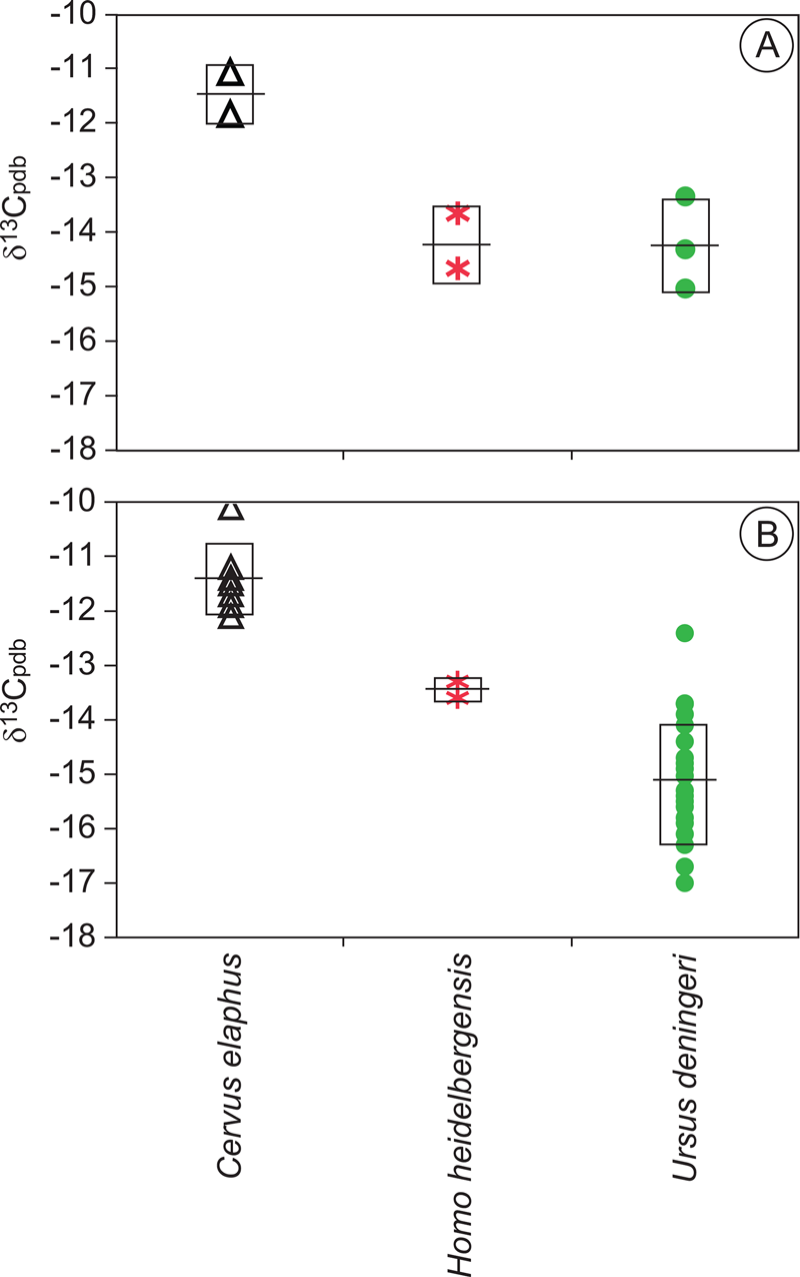
Comparison of average laser ablation carbon isotope values (A) to values obtained by the bulk sampling technique (B) for specimens analyzed by laser ablation in this study. Non-hominin bulk sampling data from [31] (Garcia Garcia et al., 2009). Line represents mean value, while boxes represent one standard deviation from mean. Results show a similar pattern between the laser ablation and bulk sampling data.

**Fig 4:**
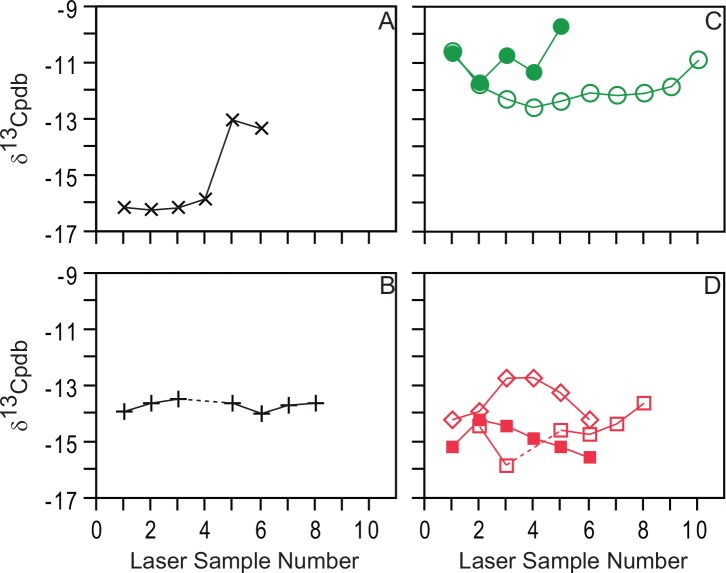
Laser ablation stable carbon isotope data for specimens analyzed in the study. A, hominin canine AT-825. B, hominin incisor AT-146. C, *Cervus elaphus* samples: open circles, molar ATA88-TGIIIa- -F21-43; closed circles, lower third molar ATA04-TD10-J21-234. D, *Ursus deningeri* samples: open squares, left lower first molar SH97-U14-137-Arcillas; closed squares, lower canine SH02-R17-Brecha; diamond, left lower first molar SH02-R/S-16/17-Arcillas. Symbols are larger than the precision (<0.3‰) for each scan.

The data from laser scans show clear isotopic differences among the species analyzed (Table 1; Figs 3A and 4). The data for the herbivores are similar in pattern to results from these fauna using the conventional bulk sampling data (Fig 3B; [31]. The most positive mean δ^13^C value for an individual was observed in *Cervus elaphus*, while the most negative individual mean value was observed in the hominin specimens. For the red deer, the range of values observed for each individual, that is, within each sampled tooth, was equal (2.0‰), and the maximum change between any two laser scans was 1.6‰. This amount of change within the tooth is not out of the range of natural variation typically observed among plants within an ecosystem, over a few seasons [55, 62, 71–73], or for a generalist herbivore capable of feeding on both open-habitat and more closed-habitat vegetation [31, 39–40]. Similarly, the greatest amount of variation in the sampled bear teeth was 2.2‰ (Sample: SH97-U14-137-Arcillas) and the maximum change observed between any two scans was only 1.4‰. Again, this change can be explained by natural variation in the ecosystem or over seasons. Thus, these bear data do not appear to suggest significant seasonal changes in ecology, as one might expect as the result of hibernation. This does not mean that the bears were not hibernating or were unaffected by hibernation. Actually, it is likely that bears sought out the Sierra de Atapuerca for hibernation [2]. For these specimens, the lack of significant changes in δ^13^C values in bears may simply indicate that hibernation had limited effect on carbon isotope values and/or that their diets did not change much across seasons. Scrutinizing the maximum and minimum values for all the laser scans of the sampled bears and/or red deer shows a difference of about 3‰ within the species (2.9‰ for red deer, 3.1‰ for bears). This likely indicates a dietary difference for one sample interval in one individual bear versus another sample interval in a different individual bear. Ecologically, this is expected as individual diets vary over time. This also makes sense paleontologically, as these individual teeth may not have developed during the same year. To understand ontogenetic changes in diet for an individual, we focus on the data within each individual tooth, not between individuals.

**Table 1:** Taxon, specimen number, element, number of laser isotope scans, and stable carbon isotope values for samples included in this study.

Species and Individual Number	Element	N	Mean δ^13^C (‰)	δ^13^C *SD* (‰)	Range (‰)
***Homo heidelbergensis***					
[1] AT-825	Canine	6	-15.1	1.5	-16.2 to -13.0
[2] AT-146	Incisor	7	-13.7	0.2	-13.9 to -13.4
***Cervus elaphus***					
[3] ATA88-TGIIIa- -F21-43	M/m	10	-11.8	0.6	-12.6 to -10.6
[4] ATA04-TD10-J21-234	m3	5	-10.8	0.8	-11.7 to -9.7
***Ursus deningeri***					
[5] SH02-R17-Brecha	c	6	-14.9	0.5	-15.5 to -14.2
[6] SH02-R/S-16/17-Arcillas	LM1	6	-13.5	0.7	-14.2 to -12.7
[7] SH97-U14-137-Arcillas	LM1	6	-14.6	0.7	-15.8 to -13.6

For the two hominin samples, different patterns emerge within the teeth (Fig 4A and 4B). The incisor (AT-146) shows very limited variability with a range of only 0.5‰. In contrast, the sampled canine (AT-825) shows a much greater range (3.2‰), which implies a change in diet or physiology. Further, for this particular canine, there is an abrupt change in δ^13^C values. Samples occurring nearer to the occlusal surface (Laser Sample 1-4), thus earlier in the life of the individual, record values around -16‰, while the two samples nearer the root (Laser Sample 5-6) record values around -13‰. Having derived from tooth enamel [46, 49–52], we suggest that the isotopic change is not the result of diagenesis. The isotopic change occurs in the middle of the tooth crown and corresponds to a crown height of approximately 4.2 mm from the neck. This change would occur at an approximate age of two to four years in modern humans [75–77], and maybe slightly younger in *H*. *heidelbergensis* [78]. Based on this age, it is possible that the isotopic change is the result of dietary changes related to weaning. The isotopic change appears to occur in a similar location on the teeth as many of the hypoplasias that were found on other SH specimens [21]. However, correlating this isotopic change directly to weaning is complicated as prior studies examining weaning through isotopic analysis have generally found that young individuals that are still nursing tend to be more positive in δ^13^C value than their parents for a particular tissue [15–18]. These studies typically focus analysis on the organic fraction of a sample (e.g., collagen) rather than the mineral fraction. In contrast, a study analyzing the mineral fraction of tooth enamel found a similar pattern to our data here [19], where tooth enamel that was formed before weaning was more negative in δ^13^C values than that formed after weaning. Additionally, weaning is, in general, a gradual process which would appear as a gradual isotopic change in tooth enamel [79–80]. This contrasts the abrupt signal observed in the laser data presented here. Regardless of whether or not the carbon isotope change observed in AT-825 is specifically related to weaning, the laser GC/IRMS data appear to provide an additional route to investigate changes in diet and resource use as a possible indicator of significant physiological change. Identification of additional individuals that show significant isotopic changes within the SH hominin population may call for a reassessment of the environmental and/or ecological conditions in which these humans lived. This idea should be explored further in a future study.

### Comparison of Isotope Sampling Techniques

**Fig 5:**
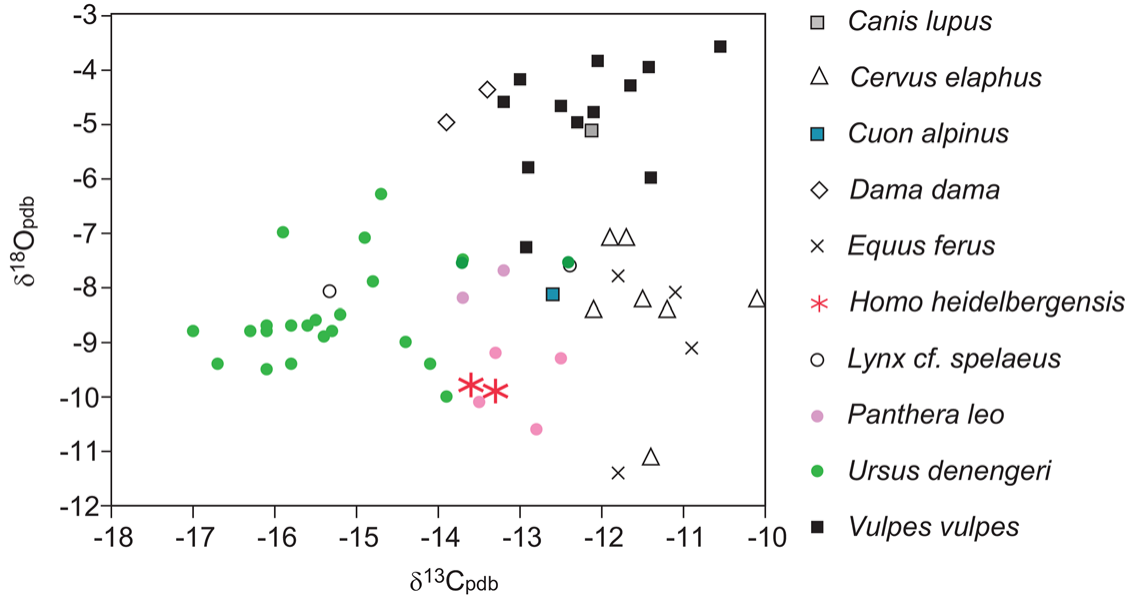
Comparison of stable carbon and oxygen isotope data of hominins from this study with previously published data of contemporaneous fauna from the Middle Pleistocene of the Sierra de Atapuerca [31] (Garcia Garcia et al., 2009).

Along with the laser data, each of the individuals analyzed in this study has been sampled and analyzed through the bulk sampling technique ([31], this study). Garcia-Garcia et al. (2009) [31] focused on examining bulk sampled non-human fauna of Atapuerca-Faunal Unit 6 and found paleoecological differences among the analyzed ungulates and carnivorans [31]. Focusing here on the hominins, comparing the bulk sampled isotope values obtained in this study for the human specimens AT-146 and AT-825 to the data in Garcia-Garcia et al. (2009) [31] shows that the values occur nearest to hypercarnivores, the lions (*Panthera leo*) and cuon (*Cuon alpinus*) (Fig 5) [31]. Although this limited sample size for the SH hominin necessitates caution be taken in making an assessment of paleodiet, it is intriguing that these humans appear isotopically similar to contemporaneous large predators, comparable to results observed for Neanderthals [31, 68, 81–82].

Having both the bulk sampled and laser ablation isotope values, including vertical laser scans on the hominin and bear specimens, provides an opportunity to evaluate the data from laser ablation GC/IRMS to that from the more conventional bulk sampling technique. Comparing the data, it appears that the mean values combining the multiple ontogenetic laser scans on each sampled tooth are similar, although not exact, to those obtained by bulk sampling or the single vertical laser scans. Overall, the observed differences are consistent with Passey and Cerling (2006) [38], a previous laser study that also compared laser derived values from those obtained by the conventional bulk sampling methodology. Using the recent and fossil data from Passey and Cerling (2006) [38] with that obtained from this study, serial laser average–conventional isotopic offsets had a mean value (δ^13^C_laser_−δ^13^C_conv_) of -0.3‰, and a total range from -2.3‰ to 1.7‰ (Table A in S1 File) [38].

The differences that are observed are at least in some part due to where and how samples were taken as well as to the isotopic variability in the sample. The closest agreement between laser-based and conventional isotopic values was observed for specimens that showed limited isotopic heterogeneity or whose stable isotope values fluctuated closely about a mean value (e.g., TD04-J21-234, AT-146). With the traditional bulk sampling method, the area of the tooth crown nearest the root (i.e., the neck) was avoided due to the enamel being thin in that area (Fig 2), and the fear that sampling there would result in fracture of the specimen. In contrast, the laser was able to sample the tooth crown from the occlusal surface to the neck in many specimens. For AT-825, the more isotopically variable of the two hominins sampled, the bulk sample corresponded to laser scans 3-6. The average of these four laser scans (-14.5‰) is closer to the bulk sample (-13.3‰) than is the average for all the laser scans of this sample (-15.1‰; Table 2). For AT-146, which displayed little isotopic variation, the average of the laser scans (3-7) corresponding to the position of the bulk sample is identical (-13.7‰) to the average for all laser scans and nearly identical to the vertical scan value (-13.6‰).

**Table 2:** Comparison of stable carbon isotope values obtained from laser ablation including the multiple serial laser scans and single vertical scan (this study), and the more traditional single bulk sampling method ([32]; this study).

Species and Individual Number	Serial Laser Mean δ^13^C (‰)	Bulk Drill. δ^13^C (‰)	Vert. Laser δ^13^C (‰)	Differ. (Serial–Bulk)	Differ. (Serial–Vert)	Differ. (Vert–Bulk)
***Homo heidelbergensis***						
[1] AT-825	-15.1	-13.3	-14.5	-1.8	+0.6	+1.2
[2] AT-146	-13.7	-13.6	-13.6	-0.1	+0.1	0
***Cervus elaphus***						
[3] ATA88-TGIIIa- -F21-43	-11.8	-11.4	N/A	-0.4	—	—
[4] ATA04-TD10-J21-234	-10.8	-10.9	N/A	+0.1	—	—
***Ursus deningeri***						
[5] SH02-R17-Brecha	-14.9	-15.9	-15.3	+1.0	-0.4	-0.6
[6] SH02-R/S-16/17-Arcillas	-13.5	-15.8	-13.4	+2.3	+0.1	-2.4
[7] SH97-U14-137-Arcillas	-14.6	-15.4	-13.9	-0.8	+0.7	-1.5

As observed in the offset between the average serial laser scans to the conventional bulk sampling, comparing the vertical laser scan taken on the bear and hominin teeth to the average serial laser scans or the bulk sample from the same tooth shows a limited offset. The vertical laser scan–average serial laser scan isotopic offsets had a mean value (δ^13^C_vertical_−δ^13^C_laser_) of +0.2‰, and a total range from -0.4‰ to 0.7‰, while the vertical laser scan–conventional bulk sample isotopic offsets had a mean value (δ^13^C_vertical_−δ^13^C_conv_) of +0.7‰, and a total range from -0.8‰ to 2.4‰. In order to combine the data using the different sampling techniques utilized here (i.e., laser and conventional bulk sampling) it is important that sampling strategies, particularly the location on the tooth, be replicated.

## Conclusions

Previous analysis of hominin fossils from the middle Pleistocene Sima de los Huesos locality of northern Spain showed a limited percentage of hypoplasias among the hominin population at this locality, possibly indicating that the hominins were well adapted to the environment [20–21]. The majority of hypoplasias that were identified typically occurred on young individuals, possibly related to weaning. Laser ablation GC/IRMS was used to analyze stable carbon isotope values in the teeth of hominins, red deer, and bears from this locality in order to explore the possibility that isotopic analysis could be used as an additional or alternative technique to distinguish periods of significant physiological changes in the SH hominin population. Most of the specimens analyzed displayed carbon isotope values within the normal range of variation observed across populations or over seasons. However, the sampled hominin canine had δ^13^C values that indicated a clear change in diet. Based on mineralization rates of modern humans, this change in diet is estimated to have occurred in the individual between the ages of two and four. An additional comparison of the laser data to that obtained from the more traditional bulk sampling methodology suggests that in order to combine the data from the two techniques strict controls are needed to ensure replicating exactly where samples are obtained on a tooth. From the data it appears that the analysis of δ^13^C values can be used as an additional tool to identify periods of physiological change in populations.

## Supporting Information

S1 File Additional information and data related to the laser ablation analysis of hominins from the Sima de los Huesos, Spain. Additional background information is provided regarding tooth enamel formation and diagenesis. Also provided are data comparing average laser ablation stable isotope values to bulk sampling (Table A) as well as all original stable isotope values from laser ablation (Appendix A) and CO_2_ standards (Appendix B).
(DOCX)
