# Supplementary material for: Exploring the Potential of Laser Ablation Carbon Isotope Analysis for Examining Ecology during the Ontogeny of Middle Pleistocene Hominins from Sima de los Huesos (Northern Spain)
Source: PLoS One. 2015 Dec 16;10(12):e0142895. doi: 10.1371/journal.pone.0142895 (PMC4686013; doi:10.1371/journal.pone.0142895)
Supplement: S1 File — Additional background information is provided regarding tooth enamel formation and diagenesis. Also provided are data comparing average laser ablation stable isotope values to bulk sampling (Table A) as well as all original stable isotope values from laser ablation (Appendix A) and CO2 standards (Appendix B). (DOCX) [file pone.0142895.s001.docx]

**S1 File. Additional information and data related to the laser ablation analysis of hominins from the Sima de los Huesos, Spain.** Additional background information is provided regarding tooth enamel formation and diagenesis. Also provided are data comparing average laser ablation stable isotope values to bulk sampling (Table A) as well as all original stable isotope values from laser ablation (Appendix A) and CO_2_ standards (Appendix B).

**Tooth Enamel Formation and Stable Isotope Values**

Tooth enamel formation, amelogenesis, occurs in a two-step process, enamel matrix deposition followed by maturation. The timing of these two steps influences the stable carbon and oxygen isotope values incorporated into the mineral that is ultimately sampled and analyzed. During the first step, enamel matrix is deposited by specialized cells, called ameloblasts. This matrix is protein rich but mineral poor, consisting of only about 20% mineral. During maturation, the mineral content of the enamel increases, but this process is gradual. Because the steps of amelogenesis are offset, the analyzed isotope ratio, by bulk sampling, serial sampling through drilling, or laser ablation, represents the time-averaged ratio for both the matrix deposition and mineralization processes.

**Diagenesis**

Diagenesis, particularly related to changes in isotopic values of the tooth enamel, was a concern for early geochemical studies examining fossils, particularly bone apatite (Schoeninger and DeNiro 1982). Tooth enamel is analyzed for stable isotope values within this study. While the effects of diagenesis may be a concern when examining certain fossil tissues for stable isotope values (i.e., bone apatite), several studies have shown that tooth enamel reliably reflects original stable carbon isotope values Krueger, 1991; Wang and Cerling, 1994; Koch et al., 1997; Lee‑Thorp and Sponheimer, 2003; Zazzo et al., 2004).

**Table A.** **Comparison of stable carbon isotope values for the averages of laser serial scans to bulk sampling.** Data from this study and Passey and Cerling (2006).

| **Sample No.** | **δ^13^C laser serial scan mean (‰)** | **δ^13^C conventional bulk sample (‰)** | **δ^13^C _laser-conv_** |
| --- | --- | --- | --- |
| AT-825^A^ | -15.1 | -13.3 | -1.8 |
| AT-146^A^ | -13.7 | -13.6 | -0.1 |
| ATA88-TG11-GSU12-F21-43^A^ | -11.8 | -11.4 | -0.4 |
| TD04-J21-234^A^ | -10.8 | -10.9 | 0.1 |
| SH02-R17-Brecha-(Z:35-48)^A^ | -14.9 | -15.9 | 1.0 |
| SH02-R/S-16/17-Arcillas-(Z:68-93)^A^ | -13.5 | -15.8 | 2.3 |
| SH97-U14-137-Arcillas^A^ | -14.6 | -15.4 | 0.8 |
| Arg2002-23^B^ | -9.6 | -8.6 | -1.0 |
| Arg2002-24^B^ | -8.8 | -8.9 | 0.1 |
| Arg2002-26^B^ | -4.3 | -2.9 | -1.4 |
| Arg2002-28^B^ | -8.0 | -8.9 | 0.9 |
| Arg2002-32^B^ | -11.5 | -11.3 | -0.2 |
| Arg2002-33^B^ | -10.4 | -10.3 | -0.1 |
| Arg2002-53^B^ | -11.0 | -11.0 | 0.0 |
| Arg2002-7^B^ | -6.0 | -5.5 | -0.5 |
| Arg2002-9^B^ | -7.2 | -6.6 | -0.6 |
| IMNH 19001^B^ | -11.7 | -10.9 | -0.8 |
| Loth 61 L2^B^ | -1.5 | 0.0 | -1.5 |
| SH 15751^B^ | -3.5 | -2.5 | -1.0 |
| UNSM 122041^B^ | -10.4 | -8.7 | -1.7 |
| USNM 4220^B^ | -8.6 | -9 | 0.4 |
| Beta Idi1^C^ | -10.9 | -12.3 | 1.4 |
| Beta ldP2^C^ | -6.4 | -7.5 | 1.1 |
| CRF ODOC1-p4^C^ | -15.0 | -13.7 | -1.3 |
| Epsilon Idi1^C^ | -12.6 | -12.4 | -0.2 |
| K00-AB-302p4.1^C^ | -13.4 | -12.7 | -0.7 |
| K01-LAI-218.p1^C^ | -6.2 | -5.3 | -0.9 |
| K01-LAI-224.p1^C^ | -11.9 | -10.8 | -1.1 |
| K01-LAI-235.p1^C^ | -0.3 | 1.2 | -1.5 |
| MA-A-11-V11-04^C^ | -14.3 | -14.2 | -0.1 |
| MA-A-20-V11-04^C^ | -12.7 | -11.9 | -0.8 |
| RRMC1.1^C^ | -12.4 | -13.8 | 1.4 |

^A^Data from this study, ^B^Fossil data from Passey and Cerling (2006), ^C^Modern data from Passey and Cerling (2006).

**Appendix A. Stable carbon isotope values for serial scans and vertical scans of individuals sampled within this study.**

| **Sample No.** | **Species** | **Sample ID** | **δ^13^C** |
| --- | --- | --- | --- |
| 08RSFL-016, scan 1 | *Homo heidelbergensis* | AT-825 | -16.1 |
| 08RSFL-016, scan 2 | *Homo heidelbergensis* | AT-825 | -16.2 |
| 08RSFL-016, scan 3 | *Homo heidelbergensis* | AT-825 | -16.1 |
| 08RSFL-016, scan 4 | *Homo heidelbergensis* | AT-825 | -15.8 |
| 08RSFL-016, scan 5 | *Homo heidelbergensis* | AT-825 | -13.0 |
| 08RSFL-016, scan 6 | *Homo heidelbergensis* | AT-825 | -13.3 |
| 08RSFL-016, scan 7,vertical | *Homo heidelbergensis* | AT-825 | -14.5 |
|  |  |  |  |
| 08RSFL-017, scan 1 | *Homo heidelbergensis* | AT-146 | -13.9 |
| 08RSFL-017, scan 2 | *Homo heidelbergensis* | AT-146 | -13.6 |
| 08RSFL-017, scan 3 | *Homo heidelbergensis* | AT-146 | -13.4 |
| 08RSFL-017, scan 4 | *Homo heidelbergensis* | AT-146 | -15.5^A^ |
| 08RSFL-017, scan 5 | *Homo heidelbergensis* | AT-146 | -13.6 |
| 08RSFL-017, scan 6 | *Homo heidelbergensis* | AT-146 | -13.9 |
| 08RSFL-017, scan 7 | *Homo heidelbergensis* | AT-146 | -13.7 |
| 08RSFL-017, scan 8 | *Homo heidelbergensis* | AT-146 | -13.6 |
| 08RSFL-017, scan 9 | *Homo heidelbergensis* | AT-146 | -14.8^A^ |
| 08RSFL-017, scan 10, vertical | *Homo heidelbergensis* | AT-146 | -13.6 |
|  |  |  |  |
| 08RSFL-015, scan 1 | *Ursus deningeri* | SH-02 | -15.2 |
| 08RSFL-015, scan 2 | *Ursus deningeri* | SH-02 | -14.2 |
| 08RSFL-015, scan 3 | *Ursus deningeri* | SH-02 | -14.4 |
| 08RSFL-015, scan 4 | *Ursus deningeri* | SH-02 | -14.9 |
| 08RSFL-015, scan 5 | *Ursus deningeri* | SH-02 | -15.2 |
| 08RSFL-015, scan 6 | *Ursus deningeri* | SH-02 | -15.5 |
| 08RSFL-015, scan 7, vertical | *Ursus deningeri* | SH-02 | -15.3 |
|  |  |  |  |
| 08RSFL-19, scan 1 | *Ursus deningeri* | SH-02 R/S.16/17 | -14.2 |
| 08RSFL-19, scan 2 | *Ursus deningeri* | SH-02 R/S. 16/17 | -13.9 |
| 08RSFL-19, scan 3 | *Ursus deningeri* | SH-02 R/S. 16/17 | -12.7 |
| 08RSFL-19, scan 4 | *Ursus deningeri* | SH-02 R/S. 16/17 | -12.7 |
| 08RSFL-19, scan 5 | *Ursus deningeri* | SH-02 R/S. 16/17 | -13.2 |
| 08RSFL-19, scan 6 | *Ursus deningeri* | SH-02 R/S. 16/17 | -14.2 |
| 08RSFL-19, scan 7, vertical | *Ursus deningeri* | SH-02 R/S. 16/17 | -13.4 |
|  |  |  |  |
| 08RSFL-018, scan 1 | *Ursus deningeri* | SH-97 U14-137 | -13.0 |
| 08RSFL-018, scan 2 | *Ursus deningeri* | SH-97 U14-137 | -14.4 |
| 08RSFL-018, scan 3 | *Ursus deningeri* | SH-97 U14-137 | -15.8 |
| 08RSFL-018, scan 4 | *Ursus deningeri* | SH-97 U14-137 | -16.1^A^ |
| 08RSFL-018, scan 5 | *Ursus deningeri* | SH-97 U14-137 | -14.6 |
| 08RSFL-018, scan 6 | *Ursus deningeri* | SH-97 U14-137 | -14.7 |
| 08RSFL-018, scan 7 | *Ursus deningeri* | SH-97 U14-137 | -14.3 |
| 08RSFL-018, scan 8 | *Ursus deningeri* | SH-97 U14-137 | -13.6 |
| 08RSFL-018, scan 9, vertical | *Ursus deningeri* | SH-97 U14-137 | -13.9 |
|  |  |  |  |
| 089RSFL-020, scan 1 | *Cervus elaphus* | ATA 88 TG11 GSU 12 F11-.43 | -10.6 |
| 089RSFL-020, scan 2 | *Cervus elaphus* | ATA 88 TG11 GSU 12 F11-.43 | -11.7 |
| 089RSFL-020, scan 3 | *Cervus elaphus* | ATA 88 TG11 GSU 12 F11-.43 | -12.2 |
| 089RSFL-020, scan 4 | *Cervus elaphus* | ATA 88 TG11 GSU 12 F11-.43 | -12.6 |
| 089RSFL-020, scan 5 | *Cervus elaphus* | ATA 88 TG11 GSU 12 F11-.43 | -12.3 |
| 089RSFL-020, scan 6 | *Cervus elaphus* | ATA 88 TG11 GSU 12 F11-.43 | -12.0 |
| 089RSFL-020, scan 7 | *Cervus elaphus* | ATA 88 TG11 GSU 12 F11-.43 | -12.1 |
| 089RSFL-020, scan 8 | *Cervus elaphus* | ATA 88 TG11 GSU 12 F11-.43 | -12.1 |
| 089RSFL-020, scan 9 | *Cervus elaphus* | ATA 88 TG11 GSU 12 F11-.43 | -11.8 |
| 089RSFL-020, scan 10 | *Cervus elaphus* | ATA 88 TG11 GSU 12 F11-.43 | -10.9 |
|  |  |  |  |
| 08RSFL-021, scan 1 | *Cervus elaphus* | ATA04 TD10 J21-234 | -10.6 |
| 08RSFL-021, scan 2 | *Cervus elaphus* | ATA04 TD10 J21-234 | -11.7 |
| 08RSFL-021, scan 3 | *Cervus elaphus* | ATA04 TD10 J21-234 | -10.7 |
| 08RSFL-021, scan 4 | *Cervus elaphus* | ATA04 TD10 J21-234 | -11.3 |
| 08RSFL-021, scan 5 | *Cervus elaphus* | ATA04 TD10 J21-234 | -9.7 |

^A^Stable isotope value not included in the analysis due to the production of smoke and/or char during ablation, which is known to cause isotopic variation.

**Appendix B. Stable carbon isotope values for all CO_2_ standards administered in this study.** Aliquots of 25uL of 3.2% CO2 were analyzed.

| **Sample No.** | **δ^13^C_CO2injection_** |
| --- | --- |
| 1 | -8.69 |
| 2 | -8.46 |
| 3 | -8.65 |
| 4 | -7.54 |
| 5 | -9.00 |
| 6 | -8.90 |
| 7 | -8.94 |
| 8 | -8.54 |
| 9 | -8.66 |
| 10 | -8.65 |
| 11 | -8.55 |
| 12 | -8.56 |
| 13 | -8.62 |
| 14 | -8.68 |
| 15 | -8.15 |
| 16 | -8.55 |
| 17 | -8.43 |
| 18 | -8.76 |
| 19 | -8.89 |
| 20 | -8.32 |
| 21 | -9.04 |
| 22 | -8.46 |
| 23 | -8.42 |
| 24 | -8.50 |
| 25 | -8.60 |
| 26 | -8.60 |
| 27 | -8.60 |

**S1 References.**

Koch PL, Tuross N, Fogel ML. The effects of sample treatment and diagenesis on the isotopic integrity of carbonate in biogenic hydroxylapatite. J Archaeol Sci. 1997;24: 417-429.

Krueger HW. Exchange of carbon with biological apatite. J Arch Sci. 1991;18: 355-361.

Lee‑Thorp JA, Sponheimer M. Three case studies used to reassess the reliabilityof fossil bone and enamel isotope signals for paleodietary studies. J Anthrop Arch. 2003;22: 208-216.

Passey BH, Cerling TE. In situ stable isotope analysis (δ^13^C, δ^18^O) of very small teeth using laser ablation GC/IRMS. Chem Geol. 2006;235: 238-249.

Schoeninger MJ, DeNiro MJ. Carbon isotope ratios of apatite from fossil bone cannot be used to reconstruct diets of animals. Nature. 1982;297: 577-578.

Wang Y, Cerling TE. A model of fossil tooth and bone diagenesis: implications for paleodiet reconstruction from stable isotopes. Palaeogeogr Palaeoclimatol Palaeoecol. 1994;107: 281-289.

Zazzo A, Lecuyer C, Mariotti A. Experimentally-controlled carbon and oxygen isotope exchange between bioapatites and water under inorganic and microbially-mediated conditions. Geochim et Cosmochim Acta. 2004;68: 1-12.
